# Normal lung sparing Tomotherapy technique in stage III lung cancer

**DOI:** 10.1186/s13014-017-0905-x

**Published:** 2017-11-06

**Authors:** Chae-Seon Hong, Sang Gyu Ju, Yong Chan Ahn, Gyu Sang Yoo, Jae Myoung Noh, Dongryul Oh, Kwangzoo Chung, Hongryull Pyo, Kwanghyun Jo

**Affiliations:** 1 Department of Radiation Oncology, Samsung Medical Center, Sungkyunkwan University School of Medicine, Irwon-Ro 81, Gangnam-Gu, Seoul, 06351 South Korea; 2 Department of Medical Device Management and Research, SAIHST, Sungkyunkwan University, Irwon-Ro 81, Gangnam-Gu, Seoul, 06351 South Korea

**Keywords:** Lung cancer, Intensity-modulated radiotherapy, Tomotherapy, Radiation pneumonitis

## Abstract

**Purpose:**

Radiation pneumonitis (RP) has been a challenging obstacle in treating stage III lung cancer patients. Beam angle optimization (BAO) technique for Tomotherapy was developed to reduce the normal lung dose for stage III non-small cell lung cancer (NSCLC). Comparative analyses on plan quality by 3 different Intensity-modulated radiation therapy (IMRT) methods with BAO were done.

**Materials and methods:**

Ten consecutive stage IIIB NSCLC patients receiving linac-based static IMRT (L-IMRT) with total 66 Gy in 33 fractions to the PTV were selected. Two additional Tomotherapy-based IMRT plans (helical beam (TH-IMRT) and static beam (TD-IMRT)) were generated on each patient. To reduce the normal lung dose, Beam angles were optimized by using complete and directional block functions in Tomotherapy based on knowledge based statistical analysis. Plan quality was compared with target coverage, normal organ sparing capability, and normal tissue complication probability (NTCP). Actual beam delivery times and risk of RP related with planning target volume (PTV) were also evaluated.

**Results:**

The best PTV coverage measured by conformity index and homogeneity index was achievable by TH-IMRT (0.82 and 1.06), followed by TD-IMRT (0.81 and 1.07) and L-IMRT (0.75 and 1.08). Mean lung dose was the lowest in TH-IMRT plan followed by TD-IMRT and L-IMRT, all of which were ≤20 Gy. TH-IMRT plan could significantly lower the lung volumes receiving low to medium dose levels: V_5~30_ when compared to L-IMRT plan; and V_5~20_ when compared to TD-IMRT plan, respectively. TD-IMRT plan was significantly better than L-IMRT with respects to V_20_ and V_30_ and there was no significant difference with respect to V_40_ among three plans. The NTCP of the lung was the lowest in TH-IMRT plan, followed by TD-IMRT and L-IMRT (6.42% vs. 6.53% vs. 8.11%). Beam delivery time was the shortest in TD-IMRT plan followed by L-IMRT. As PTV length increased, NTCP and Mean lung dose proportionally increased significantly in all three plans.

**Conclusion:**

Advantageous profiles by TH-IMRT could be achieved by BAO by complete and directional block functions. Current observation could help radiation oncologists to make wise selection of IMRT method for stage IIIB NSCLC.

## Introduction

Over one-third of non-small cell lung cancer (NSCLC) patients are diagnosed at stage III. These have usually been the ideal candidates for high dose radiation therapy (RT) with concurrent chemotherapy [1–7]. In order to achieve improved local control and survival, escalation of RT dose should be realized, which, however, is frequently associated with toxicity risk, particularly under concurrent chemotherapy setting. Therefore, it is necessary to limit RT dose to volume of normal organs including the spinal cord, lung, heart and esophagus. Radiation pneumonitis (RP) is the most challenging obstacle and sparing of as much lung volume as possible is very important [8, 9].

The normal lung volume that receives 20 Gy or higher (V_20_) has been used as an important indicator to predict the risk of symptomatic RP [10]. Efforts to limit V_20_ below 30%, while ensuring conformal dose to the target, however, have often been difficult by conventional three-dimensional conformal RT (3D-CRT) technique. Intensity-modulated RT (IMRT), through the inverse planning technique, can provide better target coverage and more sparing of the surrounding normal organs. IMRT has resulted in better clinical outcomes than 3D-CRT in treating stage III NSCLC patients, by dose escalation with more favorably limited V_20_ constraint [11–14]. By virtue of the beam delivery method that uses a large number of beamlets, IMRT is well known to increase the lung volume receiving low dose level of 5 Gy (V_5_), which has also proved to be closely related to the RP risk. V_5_ has recently received an high level attention as an important indicator in evaluating the RT plan quality and a few reports recommend to keep V_5_ below 60~65% in the patients undergoing RT with concurrent chemotherapy [12, 15]. Though with a few advantages, routine application of IMRT in treating stage III NSCLC patients has been a challenge [16–19]. Tomotherapy, a volumetric modulated arc therapy (VMAT), is a radiation beam delivery system that combines IMRT with a helical beam delivery technique (TomoHelical IMRT [TH-IMRT], Accuray, WI, USA) with a megavoltage CT (MVCT) capability, enabling image-guided radiation therapy [20]. While excellent dosimetric benefits have been noted with TH-IMRT for various disease sites, [21–23] it has not been widely investigated or accepted for the treatment of stage III NSCLC [24, 25].

Different dose profiles are achieved within and around the target, depending on dose constraints and beam delivery methods. Classic IMRT used to put as many equally spaced beams as possible. Plan quality comparisons when using 5, 7 and 9 beams in L-IMRT were done following multi-objective function iteration to determine optimal beam angles by Liu et al., which showed that optimal determination of beam angles was more important than simple increase of beam numbers to achieve better plan quality [26]. Beam angle optimization (BAO), however, is not an easy task under 360 degrees full arc rotation. For beam angle controlling, complete and directional block functions were developed on the individual basis and incorporated into the treatment planning system (TPS) of Tomotherapy [27, 28]. The incoming beams were to be arranged so that the complete blocks did not allow any beams to pass through them at all, while the directional blocks allowed beams only if they entered PTV first [27, 28].

Linear accelerator has evolved to perform dynamic “volumetric arc” beam delivery with full arc rotation [29, 30]. On the contrary, Tomotherapy has evolved to enable static beam delivery (TomoDirect-IMRT, TD-IMRT) to decrease integral dose [23, 31]. Various types of advanced treatment techniques were employed to reduce normal lung dose, but it is still a major barrier for the RT of stage III NSCLC.

In addition, there are many patient dependent factors that affect the normal lung dose in treatment planning for stage III NSCLC. Understanding of important factors, which are closely related to normal lung dose, is very important to predict complication and select treatment technique for better clinical choice. However, factor analysis related to normal lung dose and treatment technique based on geometrical information of PTV and OARs was not clearly given for RT of stage III NSCLC. We developed a new BAO technique by employing complete and directional block function for Tomotherapy of stage III NSCLC and performed comparative analyses on plan quality with L-IMRT.

## Methods

### Patient selection, simulation, and contouring

**Fig. 1:**
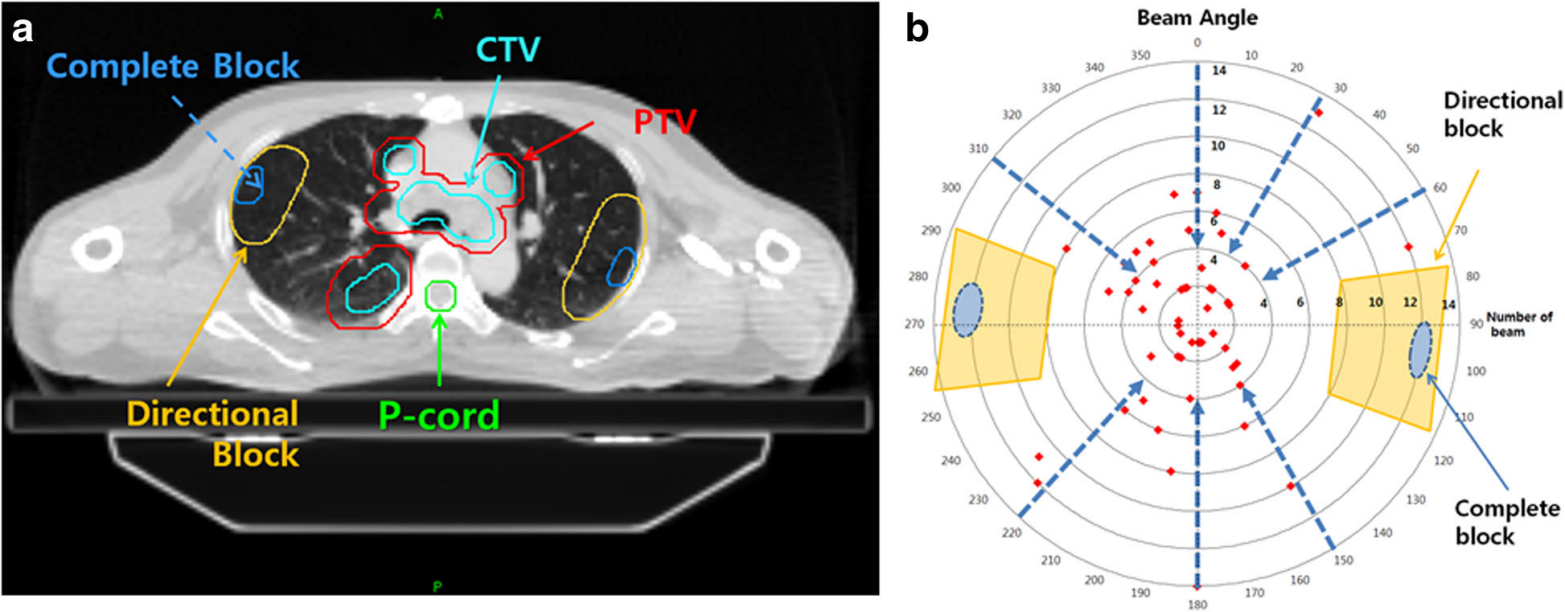
In order to lower dose to lung, complete and directional blocks were delineated within lung (**a**). These were located as from PTV as possible, and complete blocks did not allow any beams to pass through them, while directional blocks allowed beams only if they entered PTV first. In order to generate the principles of delineating the complete and directional block, authors collected and analyzed the data of beam arrangements (red dots) in L-IMRT that were actually applied to 59 N3-IIIB NSCLC patients at our institute (**b**). Dotted arrows represented the most used beam angles for L-IMRT

Authors selected ten consecutive patients having N3-IIIB NSCLC by virtue of low cervical lymph node involvement, between March 2012 and November 2013. All received definitive high dose L-IMRT (Novalis Tx®, Varian, USA) concurrent with weekly chemotherapy (Table 1). All were immobilized by individually customized cradle with foaming material in the supine position before simulation. Four-dimensional CT (4D–CT) was obtained using respiration management system (RPM®; Varian, USA), which was segmented into 10 respiratory phases, and all image sets were transferred into TPS (Pinnacle3®, version 9.2; Philips Medical System, USA). With reference to all available clinical information, the gross tumor volume (GTV) on each respiratory phase was delineated to generate the internal target volume (ITV). The clinical target volume (CTV) was generated by expansion of ITV with 5 mm margins in all directions, which was modified so that the expanded ITV did not exceed the actual anatomic boundaries such as the bone (spine, rib), tracheobronchial cartilage, chest wall, and great vessels. The planning target volume (PTV) was generated by expansion of CTV with 5 mm margin (Fig. 1). The organs at risk (OARs) were delineated including the spinal cord, normal lungs (both lung – PTV), heart and esophagus. The planning volume for the spinal cord (P-cord) was generated by adding 5 mm margin to the actual spinal cord, which was optionally reduced to 3 mm if the PTV was very close to the spinal cord.

**Table 1:** Patients’ characteristics

	Gender	Age	Primary tumor	Low neck involvement	Clinical stage	Histology
1	Female	72 years	LUL	Ipsilateral	T1 N3	Adenocarcinoma
2	Female	52 years	RLL	Ipsilateral	T1 N3	Adenocarcinoma
3	Male	58 years	RLL	Ipsilateral	T2 N3	Squamous
4	Male	49 years	RUL/RLL	Ipsilateral	T4 N3	Adenocarcinoma
5	Male	65 years	RUL	Ipsilateral	T3 N3	Adenocarcinoma
6	Male	44 years	LLL	Ipsilateral	T1 N3	Adenocarcinoma
7	Male	67 years	LLL	Contralateral	T2 N3	Squamous
8	Male	58 years	LLL	Contralateral	T2 N3	Squamous
9	Male	64 years	RUL	Bilateral	T2 N3	Adenocarcinoma
10	Male	49 years	RLL	Bilateral	T2 N3	Adenocarcinoma

*RUL* right upper lobe, *RLL* right lower lobe, *LUL* left upper lobe, *LLL* left lower lobe

### Generation of IMRT plans and beam angle optimization

For dosimetric comparison, three different IMRT plans on each patient were generated. All patients were actually treated by linear accelerator-based single isocenter step and shoot L-IMRT concurrent with weekly chemotherapy using Novalis Tx®,(Varian, USA). The numbers of static beams in L-IMRT plans were six in eight patients, five in one, and seven in one, and the same beam angles were used in TD-IMRT plans. Beam angles were optimized on trial and error basis based on BAO by Liu et al. [26].

Two additional Tomotherapy®-based IMRT plans, TH-IMRT and TD-IMRT, were generated on each patient. In order to help determine optimized beam angles through complete and directional blocks functions, pseudo-OARs (complete and directional block) within the lung were delineated so that they were located as far from PTV as possible (Fig. 1a). Because the shape, size and geometric relations between the CTV and OARs were not the same among patients, application of the pseudo-OARs in the same way was not possible. In order to generate the principles of delineating the pseudo-OARs based on knowledge based statistical analysis, authors collected and analyzed the data of beam arrangements in L-IMRT that were actually applied to 59 N3-IIIB NSCLC patients at our institute (red dots in Fig. 1b). The ideal beam directions (dotted arrows in Fig. 1b) that coincided with the method proposed by Liu et al. [26]. Pseudo-OARs should not be hit by any incoming beams and should be ≥1.5 cm from the closest PTV margin. Principally pseudo-OARs are to be delineated on both sides, but could be omitted on one side where the distance from the closest PTV was less than 1.5 cm. Circular directional block of 5 cm radius and complete block of 1.5 cm radius were to be located as far from the PTV as possible along the superior-inferior dimension of the PTV. The size and shape of the pseudo-OARs were modified considering the PTV location and the optimized dose profiles.

The angles of incoming beams in all three plans were optimized and determined according to the same rules policy. In both TH-IMRT and TD-IMRT plans, field width of 2.5 cm, modulation factor of 2.0, and pitch of 0.287 were used to avoid the thread effect [32]. In all three plans, 6-MV photon beams were used and dose calculation was done using the collapsed-cone convolution algorithm [33, 34].

### Dose constraints and optimization

All three plans followed the internal guideline on the dose constraints, which were stricter than RTOG protocol 0617 (Table 2) [35]. Two constraints were set at the highest priority level: 95% of PTV volume should receive at least 100% of the prescription dose (66 Gy/33 fractions; D_95_ ≥ 66 Gy); and the maximum dose to P-cord should not exceed 45 Gy (D_max_ < 45 Gy). In order to achieve as homogenous dose distribution as possible within and around the PTV, 99% of the PTV volume should receive at least 93% of the prescription dose (D_99_ ≥ 61.38 Gy) and the volume receiving ≥110% of the prescribed dose (72.6 Gy) should not be greater than 1 cm^3^ in total volume if within the PTV and/or in contiguous volume if outside the PTV (V_72.6 Gy_ ≤ 1 cm^3^).

**Table 2:** Dose constraints for inverse planning

Priority	Structure	Constraints
1	PTV	D_95_ ≥ 66 Gy (100%)
		D_99_ ≥ 61.38 Gy (93%)
		V_72.6 (110%)_ ≤ 1 cm^3^
1	P-cord^a^	D_max_ ≤ 45 Gy
2	Normal lung (both lung - PTV)	D_mean_ ≤ 20 Gy
		V_5_ ≤ 65%
		V_10_ ≤ 45%
		V_20_ ≤ 35%
3	Heart	V_40_ < 100%
		V_45_ < 66%
		V_60_ < 33%
4	Esophagus	D_mean_ ≤ 34 Gy

*D*_*V*_ D dose delivered to V% of organ volume, *V*_*D*_ absolute or percentage of organ volume receiving D Gy or higher, *D*_*max*_ maximum dose, *D*_*mean*_ mean dose, *OARs* organ at risks

^a^P-cord means the planning volume for the spinal cord which was generated by adding 3~5 mm margin to the actual spinal cord

The constraints at the second priority level were to limit the radiation dose to the lungs: the average lung dose (D_mean_) should not exceed 20 Gy; and the lung volume receiving 5 Gy (V_5_), 10 Gy (V_10_) and 20 Gy (V_20_) should not exceed 65, 45 and 35% of normal lung volume, respectively. The same dose constraints were applied to the pseudo-OARs.

The constraints at the third and fourth priority levels were to limit the dose to the heart and esophagus, respectively. The constraints at the third level were to limit the radiation dose to the heart: the heart volume receiving 40 Gy (V_40_), 45 Gy (V_45_) and 60 Gy (V_60_) should not exceed 100, 66 and 33% of the heart volume, respectively. The lowest level constraint was to limit the dose to the esophagus: D_mean_ to the esophagus should not exceed 34 Gy.

For each plan, the same number of iteration was used during dose optimization process. During inverse planning, once PTV constraints were reached, the optimization was continued to reduce the doses to OARs until the iteration limit while maintaining PTV dose. For dose comparison, all plans were normalized to cover 95% of the PTV with the prescription dose (66 Gy).

### Evaluation of IMRT plans

All data including calculated dose and contour information of three IMRT plans on each patient were transferred to MIM Maestro® (MIM Software Inc., USA) using the DICOMRT protocol, and objective comparisons of the dose and volume parameters were performed. To evaluate the PTV dose coverage, Conformity index (CI) and Homogeneity index (HI) were used. CI is the ratio of the prescription volume to the PTV, [36] and was calculated as the equation below:

1 $$ \mathrm{CI}=\frac{\ {PTV}_{PIV}^{\kern1.75em 2}}{PTV\times PIV\kern0.5em } $$

The PTV_PIV_ is the PTV encompassed within the volume covered by the prescription isodose surface (PIV). CI value of 1 means a perfect conformation, where the prescription isodose is identical to the target volume, and better conformity is achieved as CI value approaches 1.

HI is an indication of the dose uniformity within the PTV, defined as a ratio the dose delivered to 5% and 95% of the PTV volumes, respectively [37]. HI value of 1 is the ideal value that indicates a uniform dose distribution within the PTV.

2 $$ \mathrm{HI}=\frac{{\mathrm{D}}_5}{D_{95}} $$

For comparative evaluation of radiobiological effects on normal organs including the lung, spinal cord, heart and esophagus, the normal tissue complication probability (NTCP) using the Kutcher-Burman histogram reduction scheme in conjunction with the Lyman model [38–43] was used by applying the same calculation parameters that used by Song [43]. The comparison of NTCP’s was possible because the dose calculation algorithms were the same between the TPS (collapsed-cone convolution algorithm) [44, 45]. In addition, several relevant dosimetric parameters on these organs were used for comparisons: V_5_, V_10_, V_15_, V_20_, V_30_, V_40_ and D_mean_ of the lungs; D_max_ to P-cord; V_45_ and V_60_ of the heart; and V_50_ and V_60_ of the esophagus, respectively.

The actual beam delivery times by each plan, defined as the time from the first beam-on till the last beam-off, were measured to compare the machine workloads, which, in turn, could be used in patient throughput estimation, assuming that the times needed for patient setup before the first beam-on were equivalent to each other.

In order to evaluate the impacts of PTV-related factors on the risk of lung toxicity, the superior to inferior length of PTV (PTV length) and PTV overlapping with the normal lung (PTV ∩ Lung), in addition to PTV itself, were calculated.

The Wilcoxon signed-rank test and the Bonferroni correction (SAS version 9.4, SAS Institute, Cary, NC, USA) were used to compare the dosimetric parameters and the beam delivery time between plans one by one. The Spearman correlation test was used to determine the association between PTV-related factors and the dosimetric parameters and NTCP of the lung. Two-tailed *p* value <0.05 was considered statistically significant.

## Results

### Treatment plan evaluation

**Fig. 2:**
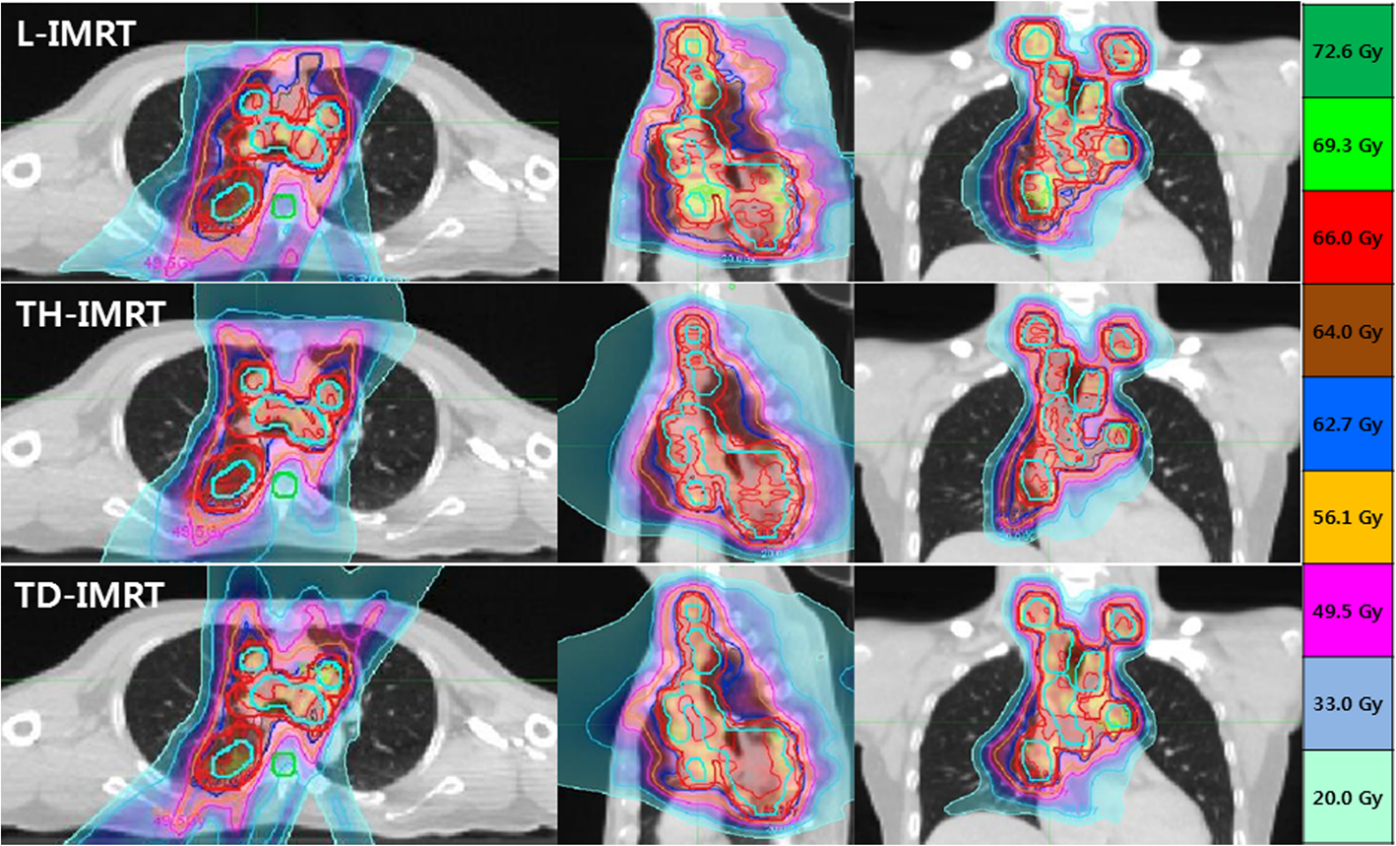
Isodose distribution of an example case in axial, sagittal, and coronal sections by L-IMRT, TH-IMRT and TD-IMRT. Apparent dose distribution looked better conformed to PTV in order of TH-IMRT, TD-IMRT and L-IMRT

The comparisons of dosimetric parameters and beam delivery times by 3 IMRT methods are summarized in Table 3. The best PTV coverage measured by CI and HI was achievable by TH-IMRT (0.82 and 1.06), followed by TD-IMRT (0.81 and 1.07) and L-IMRT (0.75 and 1.08). In one by one comparison, both TH-IMRT and TD-IMRT exhibited significantly better CI and HI than L-IMRT (all *p* values were 0.006) (Fig. 2).

**Table 3:** Comparisons of dosimetric parameters and beam delivery time

Parameters		L-IMRT	TH-IMRT	TD-IMRT	*p* ^a^		
					L-IMRT vs. TH-IMRT	L-IMRT vs. TD-IMRT	TH-IMRT vs TD-IMRT
		Median (IQR)	Median (IQR)	Median (IQR)			
PTV	CI	0.75 (0.72, 0.81)	0.82 (0.79, 0.85)	0.81 (0.77, 0.83)	0.006	0.006	0.580
	HI	1.08 (1.07, 1.08)	1.06 (1.05, 1.06)	1.07 (1.05, 1.07)	0.006	0.006	0.317
P-cord	NTCP	0.82% (0.71, 0.92)	0.34% (0.23, 0.48)	0.43% (0.28, 0.55)	0.006	0.006	0.393
	D_max_	46.44 Gy (45.56, 47.21)	44.93 Gy (40.35, 45.33)	45.55 Gy (41.41, 46.68)	0.029	0.317	0.194
Normal Lung	NTCP	8.11% (4.90, 10.11)	6.42% (3.67, 7.47)	6.53% (4.95, 9.51)	0.006	1.000	0.082
	D_mean_	17.83 Gy (14.98, 19.16)	16.49 Gy (13.49, 17.30)	16.60 Gy (14.98, 18.78)	0.006	1.000	0.082
	V_5_	62.46% (54.08,71.35)	61.46% (50.92, 68.01)	65.83% (58.94, 72.58)	0.006	0.393	0.006
	V_10_	48.48% (43.71, 53.45)	43.76% (35.66, 49.68)	51.87% (45.52, 56.77)	0.006	0.967	0.006
	V_15_	40.63% (37.17, 45.77)	34.56% (28.11, 39.09)	38.81% (34.66, 45.60)	0.006	0.580	0.001
	V_20_	35.47% (30.92, 37.54)	28.02% (23.35, 31.08)	30.30% (27.79, 34.55)	0.006	0.006	0.006
	V_30_	23.99% (20.37, 26.77)	19.65% (16.30, 21.70)	19.75% (17.69, 21.23)	0.006	0.029	0.580
	V_40_	13.26% (12.07, 18.24)	13.61% (11.35, 15.94)	12.63% (11.78, 14.91)	1.000	1.000	1.000
Heart	NTCP	27.70% (23.75, 33.50)	27.24% (23.90, 33.10)	27.44% (24.07, 32.40)	1.000	1.000	1.000
	V_45_	6.71% (0.95, 21.71)	4.10% (0.20, 11.15)	4.66% (0.90, 10.06)	0.023	0.023	1.000
	V_60_	1.92% (0.04, 7.02)	1.65% (0.00, 4.25)	1.41% (0.05, 4.53)	0.117	0.047	1.000
Esophagus	NTCP	35.57% (21.88, 46.54)	33.05% (17.92, 42.89)	36.91% (22.89, 45.17)	0.252	1.000	0.082
	V_50_	41.79% (24.36, 48.55)	38.43% (18.63, 48.17)	42.77% (22.23, 49.65)	0.059	0.580	0.393
	V_60_	29.55% (10.26, 38.90)	25.28% (9.35, 39.00)	30.25% (11.75, 39.26)	0.146	1.000	0.018
Beam delivery time		8.4 min (6.98, 10.18)	10.1 min (9.05, 11.90)	7.3 min (6.23, 8.90)	0.252	0.375	0.006

*CI* conformity index, *HI* homogeneity index, *V*_*D*_ the percentage of organ volume receiving D Gy or higher, *D*_*mean*_ mean dose, *D*_*max*_ maximum dose, *NTCP* normal tissue complication probability, *IQR* interquartile range (Q1, Q3)

^a^The Wilcoxon signed rank test was used by the Bonferroni correction for multiple testing

**Fig. 3:**
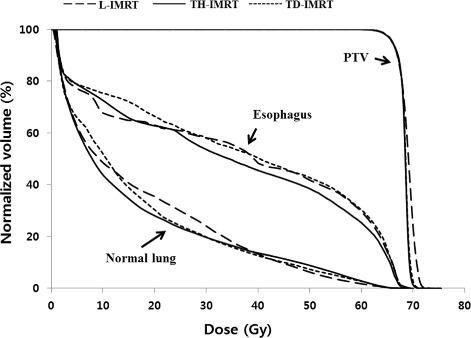
Median dose-volume histograms of all ten patients for PTV, esophagus, and normal lung by L-IMRT, TH-IMRT and TD-IMRT

The NTCP and D_max_ of the spinal cord were the lowest in TH-IMRT plan followed by TD-IMRT and L-IMRT (0.34% and 44.93 Gy vs. 0.43% and 45.55 Gy vs. 0.82% and 46.44 Gy). In one by one comparison, the NTCP’s of TH-IMRT and TD-IMRT plans were significantly lower than L-IMRT (*p* = 0.006) and D_max_ of TH-IMRT was significantly lower than L-IMRT (*p* = 0.029). The NTCP of the lung was the lowest in TH-IMRT plan, followed by TD-IMRT and L-IMRT (6.42% vs. 6.53% vs. 8.11%). In one by one comparison, the difference between TH-IMRT and L-IMRT plans was significant (*p* = 0.006). Based on the dose-volume histogram comparison, the lung volume receiving low to medium dose level was the smallest in TH-IMRT plan (Fig. 3). D_mean_ was the lowest in TH-IMRT plan followed by TD-IMRT and L-IMRT (16.49 Gy vs. 16.60 Gy vs. 17.83 Gy), all of which were ≤20 Gy and satisfied the predefined constraint, and the difference between TH-IMRT and L-IMRT plans was significant (*p* = 0.006). TH-IMRT plan was able to satisfy other three constraints on the lung (V_5_ ≤ 65%, V_10_ ≤ 45% and V_20_ ≤ 35%) and L-IMRT plan satisfied V_5_ constraint only. TH-IMRT plan could significantly lower the lung volumes receiving low to medium dose levels: V_5~30_ when compared to L-IMRT plan; and V_5~20_ when compared to TD-IMRT plan, respectively. TD-IMRT plan was significantly better than L-IMRT with respects to V_20_ and V_30_ and there was no significant difference with respect to V_40_ among three plans.

The NTCP of the heart was the lowest in TH-IMRT plan followed by TD-IMRT and L-IMRT (27.24% vs. 27.44% vs. 27.70%). V_45_ was the lowest in TH-IMRT plan followed by TD-IMRT and L-IMRT (4.10% vs. 4.66% vs. 6.71%) and V_60_ was the lowest in TD-IMRT plan followed by TH-IMRT and L-IMRT (1.41% vs. 1.65% vs. 1.92%), respectively. V_45_ of TH-IMRT and TD-IMRT plans were significantly lower than L-IMRT (*p* = 0.023) and V_60_ of TD-IMRT was significantly lower than L-IMRT (*p* = 0.047).

The NTCP of the esophagus was the lowest in TH-IMRT plan followed by L-IMRT and TD-IMRT (33.05% vs. 35.57% vs. 36.91%), however, there was no significant differences. V_50_ and V_60_ were the lowest in TH-IMRT plan followed by L-IMRT and TD-IMRT (38.43% and 25.28% vs. 41.79% and 29.55% vs. 42.77% and 30.25%). V_60_ of TH-IMRT was significantly lower than TD-IMRT (*p* = 0.018).

The beam delivery time was the shortest in TD-IMRT plan followed by L-IMRT and TH-IMRT (7.3 min vs. 8.4 min vs. 10.1 min), and that of TD-IMRT plan was significantly shorter than TH-IMRT (*p* = 0.006).

### Correlation between PTV-related parameters on lung toxicity risk

The median values of PTV, superior-inferior PTV length, and PTV volume overlapping with lung (PTV ∩ Lung) of all patients were 479 cm^3^ (interquartile range [IQR]: 417~606 cm^3^), 18.0 cm (IQR: 15.2~19.8 cm) and 5.3% (IQR: 3.5%~5.9%), respectively. The estimated impacts of PTV and PTV-related factors on the risk of RP in three different plans are summarized in Table 4. In all three plans, there was no significant correlation between RP risk and PTV itself or PTV ∩ Lung. However, as PTV length increased, NTCP and D_mean_ proportionally increased in all three plans significantly, which was the most prominent in TH-IMRT plan (*p* = 0.005). Along with increasing PTV length, V_5~20_ in L-IMRT, V_5~40_ in TH-IMRT, and V_15~20_ in TD-IMRT plans increased significantly as well (all *p* values <0.05).

**Table 4:** Comparison of correlation coefficient between parameters related with planning target volume (PTV) and lung

Parameters	Lung	L-IMRT	*p* ^c^	TH-IMRT	*p* ^c^	TD-IMRT	*p* ^c^
PTV	NTCP	−0.309	0.385	−0.261	0.467	−0.442	0.200
	D_mean_	−0.309	0.385	−0.261	0.467	−0.442	0.200
	V_5_	−0.273	0.446	−0.321	0.366	−0.406	0.244
	V_10_	−0.236	0.511	−0.224	0.533	−0.345	0.328
	V_15_	−0.273	0.446	−0.358	0.310	−0.539	0.108
	V_20_	−0.272	0.446	−0.321	0.366	−0.406	0.244
	V_30_	−0.236	0.511	−0.224	0.533	−0.345	0.328
	V_40_	−0.297	0.405	−0.152	0.676	−0.4303	0.215
PTV length^a^	NTCP	0.663	0.037	0.802	0.005	0.632	0.049
	D_mean_	0.663	0.039	0.802	0.005	0.632	0.049
	V_5_	0.657	0.039	0.687	0.028	0.523	0.121
	V_10_	0.839	0.002	0.729	0.017	0.498	0.143
	V_15_	0.851	0.002	0.802	0.005	0.644	0.044
	V_20_	0.821	0.004	0.815	0.004	0.729	0.017
	V_30_	0.547	0.102	0.711	0.021	0.553	0.097
	V_40_	0.322	0.364	0.778	0.008	0.505	0.137
PTV ∩ Lung^b^	NTCP	0.067	0.855	0.103	0.777	−0.079	0.829
	D_mean_	0.067	0.855	0.103	0.777	−0.079	0.829
	V_5_	0.103	0.777	0.152	0.676	0.115	0.751
	V_10_	0.055	0.881	−0.018	0.960	−0.139	0.701
	V_15_	0.03	0.934	−0.018	0.960	−0.127	0.726
	V_20_	−0.055	0.881	−0.042	0.907	−0.103	0.777
	V_30_	−0.018	0.960	0.115	0.751	−0.152	0.676
	V_40_	−0.03	0.934	0.273	0.446	0.018	0.960

*PTV* planning target volume, *NTCP* normal tissue complication probability, *V*_*D*_ the percentage of organ volume receiving D Gy or higher, *Dmean* mean dose

^a^PTV length means the superior-inferior length of the PTV

^b^PTV ∩ Lung means the PTV volume overlapping with the normal lung

^c^All *p* values were calculated by the Spearman correlation test

## Discussion

There are a few challenging issues of the target delineation and the interplay effects caused by respiratory and multi-leaf motions, particularly in treating lung cancer patients [17–19, 46]. Delineation of the ITV, which reflects the target motion by respiratory cycle, has been routinely performed following 4D–CT, and several previous studies have endorsed that the interplay effects could become averaged out (smearing) to the negligible level during the multi-fractioned RT course [47, 48].

Beam delivery by full arc rotation, in general, has the merit of high conformity within the deep tissues, and, at the same time, has the demerit of increased integral dose to the peripheral body parts. Because the relative electron density of the lung is around 0.3, the range of radiation in the normal lung that surrounds solid tumor becomes naturally longer than in other body parts. In high dose RT settings, coupled with low relative electron density, the lung volume receiving low to moderate dose level frequently becomes quite large in proportion to volume and superior to inferior length of target. A few reports currently recommend to keep V_5_ below 60~65% in high dose RT and concurrent chemotherapy setting [12, 15]. In this context, beam delivery from within restricted ranges, rather than full arc rotation, is essentially necessary to reduce the lung volume receiving low to moderate dose [26].

BAO can be realized in different ways by inverse planning algorithm (IPA) of the TPS and beam delivery technique. In this context, direct comparison of BAO techniques with Linear accelerator based volumetric modulated arch therapy (L-VMAT) and TH-IMRT is not an easy task because they have different IPS and beam delivery options. A few reports introduced L-VMAT techniques for treatment of stage III NSCLC, not included in this study [49, 50]. General consensus of these was full arc VMAT can increase lung volume receiving low to moderate dose in RT for stage III NSCLC. They recommended the use of partial arc VMAT or hybrid (partial arc combine with static IMRT beams) technique, which is another type of BAO for L-VMAT, to meet criteria for normal lung dose instead of full arc VMAT. Through indirect comparison with theses report, our data showed reasonable and comparable result in normal lung sparing capability.

TH-IMRT is capable to generate superior dosimetric profiles to L-IMRT by full arc helical beam delivery method, and has demonstrated favorable clinical outcomes in various disease sites [20–23]. Though there have been many clinical studies implementing IMRT in treating lung cancer, clinical use of TH-IMRT has been rather less active, mainly in fear of increased integral dose, most of which can be delivered to the normal lung [25, 51]. With the same general consensus compared to full arc VMAT, two studies using TH-IMRT without BAO reported that an increased V_5–10_ was associated with serious lung toxicity [24, 43]. Determination of optimal beam angles that can reduce the normal lung volume receiving clinically significant radiation dose is not an easy task in TH-IMRT, which deliver radiation dose through Helical beam delivery technique, rapid rotating of the gantry with table translation. Liu et al., through multi-objective optimization, proposed the beam angles confined within rather narrow ranges of anterior and posterior oblique directions, which generated the typical “butterfly-like” shape [26]. This directional blocking concept could reduce the low dose lung volume considerably, when compared to the previous reports [24, 43]. In this respect, our study is unique in that, for the first time, authors further controlled the beam angles passing through the normal lung, which was far from the target, by using the complete blocking in addition to the directional blocking function (Fig. 1) in RT for stage III NSCLC. Since the complete blocks can limit the freedom of beam angle more, and, as a result, can compromise the target coverage, it is advised to delineate this as small as possible and as far from the target as possible. Meanwhile, application of rather generous and large directional blocks outside the target may be allowed.

Though a new beam delivery method, as in TD-IMRT, has been introduced to Tomotherapy-based static IMRT, the dosimetric characteristics have not been fully addressed as of yet for treating stage III NSCLC. Furthermore, the significance of BAO policy by complete and directional block functions, which is relatively new and advanced, has not been properly evaluated, and the current study is the first comprehensive and comparative evaluation of 3 different IMRT techniques for treating stage III NSCLC.

## Conclusions

Through the dosimetric comparisons among 3 IMRT plans, a few remarkable findings could be summarized. First, CI and HI could have been significantly improved by TH- and TD-IMRT when compared to L-IMRT. Second, TD-IMRT seems to be more advantageous over L-IMRT on V_20_ and V_30_, but more disadvantageous over TH-IMRT on V_5~20_. Third, TH-IMRT only could have fulfilled all dosimetric constraints of the lung, which is contradictory to the previous belief that helical beam delivery can increase the integral dose. Authors would strongly believe that these advantageous profiles by TH-IMRT could be achieved by BAO policy employed in the current study. Fourth, beam delivery time is significantly longer by TH-IMRT than TD-IMRT. Authors would wish that the current observation together with dosimetric comparison could serve to help radiation oncologists in making wise selection of IMRT technique.

## Abbreviations

4D–CT: Four-dimensional CT
BAO: Beam angle optimization
CI: Conformity index
CTV: Clinical target volume
D_max_: Maximum dose
D_mean_: Mean dose
D_V_: D dose delivered to V% of organ volume
GTV: Gross tumor volume
HI: Homogeneity index
IMRT: Intensity-modulated radiation therapy
IPA: Inverse planning algorithm
IQR: Interquartile range (Q1, Q3)
ITV: Internal target volume
L-IMRT: Linac-based static IMRT
LLL: Left lower lobe
LUL: Left upper lobe
L-VMAT: Linear accelerator based volumetric modulated arc therapy
MVCT: Megavoltage computed tomography
NSCLC: Non-small cell lung cancer
NTCP: Normal tissue complication probability
OAR: Organs at risk
P-cord: Planning volume for the spinal cord
PTV: Planning target volume
RLL: Right lower lobe
RP: Radiation pneumonitis
RT: Radiation therapy
RUL: Right upper lobe
TD-IMRT: TomoDirect-IMRT
TH-IMRT: TomoHelical-IMRT
TPS: Treatment planning system
V_D_: Percentage of organ volume receiving D Gy or higher
VMAT: Volumetric modulated arc therapy
